# Task force Guideline of Brazilian Society of Otology – hearing loss in children – Part II — Treatment

**DOI:** 10.1016/j.bjorl.2022.11.001

**Published:** 2022-11-26

**Authors:** Vagner Antonio Rodrigues Silva, Henrique Furlan Pauna, Joel Lavinsky, Miguel Angelo Hyppolito, Melissa Ferreira Vianna, Mariana Leal, Eduardo Tanaka Massuda, Rogério Hamerschmidt, Fayez Bahmad Jr, Renato Valério Cal, André Luiz Lopes Sampaio, Felippe Felix, Carlos Takahiro Chone, Arthur Menino Castilho

**Affiliations:** a Universidade Estadual de Campinas (Unicamp), Faculdade de Ciências Médicas, Departamento de Otorrinolaringologia e Cirurgia de Cabeça e Pescoço, Campinas, SP, Brazil; b Hospital Universitário Cajuru, Departamento de Otorrinolaringologia, Curitiba, PR, Brazil; c Universidade Federal do Rio Grande do Sul (UFRGS), Departamento de Cirurgia, Porto Alegre, RS, Brazil; d Universidade de São Paulo (USP), Faculdade de Medicina de Ribeirão Preto, Departamento de Oftalmologia, Otorrinolaringologia e Cirurgia de Cabeça e Pescoço, Ribeirão Preto, SP, Brazil; e Irmandade Santa Casa de Misericórdia de São Paulo, Departamento de Otorrinolaringologia, São Paulo, SP, Brazil; f Universidade Federal de Pernambuco (UFPE), Departamento de Cirurgia, Recife, PE, Brazil; g Universidade Federal do Paraná (UFPR), Hospital de Clínicas, Departamento de Otorrinolaringologia e Cirurgia de Cabeça e Pescoço, Curitiba, PR, Brazil; h Universidade de Brasília (UnB), Programa de Pós-Graduação em Ciências da Saúde, Brasília, DF, Brazil; i Instituto Brasiliense de Otorrinolaringologia (IBO), Brasília, DF, Brazil; j Centro Universitário do Estado do Pará (CESUPA), Departamento de Otorrinolaringologia, Belém, PA, Brazil; k Universidade de Brasília (UnB), Faculdade de Medicina, Laboratório de Ensino e Pesquisa em Otorrinolaringologia, Brasília, DF, Brazil; l Universidade Federal do Rio de Janeiro (UFRJ), Hospital Universitário Clementino Fraga Filho (HUCFF), Departamento de Otorrinolaringologia, Rio de Janeiro, RJ, Brazil

**Keywords:** Hearing loss, Children, Guidelines, Screening, Diagnosis, Intervention

## Abstract

**Objectives:**

To provide an overview of the main evidence-based recommendations for the diagnosis of hearing loss in children and adolescents aged 0–18 years.

**Methods:**

Task force members were educated on knowledge synthesis methods, including electronic database search, review and selection of relevant citations, and critical appraisal of selected studies. Articles written in English or Portuguese on childhood hearing loss were eligible for inclusion. The American College of Physicians’ guideline grading system and the American Thyroid Association’s guideline criteria were used for critical appraisal of evidence and recommendations for therapeutic interventions.

**Results:**

The topics were divided into 2 parts: (1) treatment of sensorineural hearing loss: individual hearing aids, bilateral cochlear implants, cochlear implants in young children, unilateral hearing loss, and auditory neuropathy spectrum disorder; and (2) treatment of conductive/mixed hearing loss: external/middle ear malformations, ventilation tube insertion, and tympanoplasty in children.

**Conclusions:**

In children with hearing loss, in addition to speech therapy, Hearing AIDS (HAs) or implantable systems may be indicated. Even in children with profound hearing loss, both the use of HAs and behavioral assessments while using the device are important.

## Introduction

Newborn hearing screening programs have diagnosed profound sensorineural hearing loss in the first few months of life. Initiating auditory rehabilitation, including the use of Hearing AIDS (HAs) before 6 months of age has significantly improved vocabulary, speech intelligibility, general language ability, and socioemotional development.^1^, ^2^ Early Cochlear Implantation (CI) improves auditory processing skills.^3^

Children who undergo cochlear implantation early in life may achieve similar verbal language skills to those with normal hearing.^4^, ^5^ In addition, when compared to unilateral or sequential surgery, simultaneous bilateral cochlear implantation improves auditory performance, auditory pathway development in the brain and, consequently, spoken language acquisition.^6^ Cochlear implantation in the first year of life has been associated with age-appropriate language gains, while comparatively poorer developmental outcomes have been observed in children undergoing surgery later in life.^7^ After 1 year of age, hearing gains decrease as the child’s age at the time of surgery increases. Colletti et al.^8^ found evidence of additional benefits when cochlear implantation is conducted before 6 months of age.

Although age at the time of cochlear implantation may have only minor long-term effects on general language understanding, it has a lasting effect on more specific and complex skills within the domains of phonetics, expressive vocabulary, grammar, and semantics. These skills depend on the functional specialization of certain networks in the brain that are triggered by sensory input during early sensitive periods of neuronal development.^8^

The use of HAs and CIs early in life has rehabilitated many children, who were able to acquire near-normal language. The effectiveness of these interventions is influenced by factors such as maternal education level, duration of daily device use, and nonverbal intelligence.^9^

Maximizing hearing in each ear optimizes oral language development. Device options for children with bilateral sensory hearing loss include HAs, CIs, and a combination of both (bimodal stimulation).

### Unilateral sensorineural hearing

Unilateral sensorineural hearing loss affects approximately 9% of the population and is less related to aging than bilateral hearing loss. Its prevalence ranges from 0.3 to 1 per 1000 newborns. Approximately 3%–6% of school-age children have some degree of unilateral hearing loss.^10^

Children with Single-Sided Deafness (SSD) often struggle with speech perception in noise and sound localization due to lack of binaural auditory input. This outcome has been associated with the absence of binaural hearing effects, such as the squelch effect, the head shadow effect, and binaural summation. SSD has implications for children’s speech language development, cognition, and quality of life and causes psychosocial and behavioral difficulties.^11^

Spatial perception is multisensory. The auditory system plays an important role by continuously detecting sounds in the environment, helping with spatial localization. Auditory space constructs are complex and depend on the interaction with signals that dynamically change in terms of frequency spectrum, intensity, and time. The more complex the environment is, the more these signals interact and overlap.^12^

Hearing does not involve only sound detection and awareness, but also the management of these complex interactions to provide meaning to these signals. The auditory system is quite sophisticated in fully managing the acoustic information presented to the ears in dynamically changing environments. Listeners can rapidly process this information to identify acoustic stimuli, selectively attending to signals of importance while suppressing competing signals.^13^

Spatial hearing depends on the processing of monaural and binaural hearing cues. Although monaural stimulus provides important information and contributes to the ability to determine the distance of a sound source, binaural hearing plays a much larger role. The integration of acoustic information from both ears is essential for spatial hearing and provides critical information for speech processing, localization, differentiation of auditory streams, and perception of fused sounds. Binaural hearing gives rise to a wide array of auditory phenomena due to the integration and processing of differences in arrival time and intensity between the signals at the two ears. The interaural timing difference is the difference in arrival time for a stimulus to reach both ears, being greatest for low frequency signals below 1000 Hz.^14^

When a sound is presented to a listener, the ear closest to the signal of interest will detect that sound before the ear farthest from the signal. Likewise, the interaural level difference dictates that the ear closer to a stimulus will receive a more intense signal compared with the contralateral ear.^14^ The head acts as a physical barrier to sounds, resulting in an attenuation of the signal in the ear not directed at the source. The acoustic shadowing of the head varies according to signal frequency and position.^15^

Animal studies have evaluated the effects of unilateral hearing loss during development on different levels of the auditory system — morphology, connectivity, and the response properties of neurons. There was weakening of the representation of the deprived ear and a strengthening of the representation of the intact ear in the cortex.^16^

### Conductive/mixed hearing loss

Microtia and congenital aural atresia are malformations that occur in approximately 1 in 10,000–20,000 live births. They are usually unilateral, with a predilection for the right ear, and are more common in boys than in girls (2.5:1). The inner ear and its function are often preserved but can be affected in 10%–20% of cases.^17^

The mean incidence of acute otitis media is 10.8 new episodes per 100 person-years,^18^ but it has declined in high-income countries due to vaccination and adoption of guidelines with strict diagnostic indications.^19^ Treatment includes analgesics, antipyretics, and antibiotics (if necessary). Tympanostomy Tube Placement (TTP) is indicated for management of serious complications, such as mastoiditis, peripheral facial paralysis, meningitis, petrositis, sigmoid sinus thrombosis, and brain abscess.

Approximately 98.7 million people have otitis media-related hearing loss, according to the World Report on Hearing,^20^ which shows an incidence of chronic otitis media of 4.76%, approximately 30 million cases/year, and a prevalence of approximately 200 million cases globally. Of these, 22.6% occur in children under 5 years of age. Otitis media is one of the main causes of hearing loss in childhood and, although the incidence of infections decrease with age, hearing loss can persist.

Chronic suppurative otitis media leads to some degree of hearing loss in 50% of cases due to tympanic membrane perforation and changes in the ossicular chain (conductive hearing loss), hair cell damage caused by recurrent infections (sensorineural hearing loss), or mixed.^21^

## Objective

This systematic review have the purpose to provide an overview of the evidence-based recommendations for the treatment of hearing loss in children and adolescents aged 0–18 years.

## Methods

Between April 28 and 29, 2022, a task force consisting of otolaryngologists, otology specialists, Brazilian Society of Otology (Sociedade Brasileira de Otologia, SBO) directors, and some SBO members met (in person and remotely) to discuss the topic of this guideline. Each participant in this meeting was tasked with giving a 15-min evidence-based lecture on one of the suggested topics. After the lecture, the participants discussed the topic until reaching a consensus. Each author was asked to write a text with the current literature on the topic, based on evidence and containing the elements discussed during the meeting. A rapporteur prepared the final text, which was reviewed by 4 additional coauthors and the Brazilian Journal of Otorhinolaryngology (BJORL) editor.

This guideline is not intended to be a substitute for individual professional judgment. Physicians should always act and decide in a way that they believe is best for their patients, regardless of guideline recommendations. They should also operate within their scope of practice and in accordance with their training. The guidelines represent the best judgment of a team of experienced physicians addressing the scientific evidence for a given topic.

The grading system of the American College of Physicians (ACP) was used in this guideline, relating to critical appraisal and recommendations on therapeutic interventions^22^ (Table 1, Table 2). An important component of this guideline was judged to be critical appraisal of diagnostic testing studies. However, the ACP guideline grading system was not designed for this purpose.^23^, ^24^, ^25^

**Table 1:** Interpretation of the American College of Physicians’ guideline grading system (for Therapeutic Interventions).

Recommendation	Clarity of risk/benefit	Implications
Strong recommendation	Benefits clearly outweigh harms and burdens, or vice versa.	Patients: most would want course of action; a person should request discussion if an intervention is not offered.
		Clinicians: most patients should receive the recommended course of action.
		Policymakers: the recommendation can be adopted as policy in most circumstances.
Weak recommendation	Benefits closely balanced with harms and burdens.	Patients: many would want course of action, but some may not; the decision may depend on individual circumstances.
		Clinicians: different choices will be appropriate for different patients; the management decision should be consistent with patients’ preferences and circumstances.
		Policymakers: policymaking will require careful consideration and stakeholder input.
No recommendation	Balance of benefits and risks cannot be determined.	Decisions based on evidence cannot be made.

**Table 2:** Recommendations (for Therapeutic Interventions) based on strength of evidence.

Recommendation and evidence of quality	Description of supporting evidence^a^	Interpretation
Strong recommendation		
High-quality evidence	RCT without important limitations or overwhelming evidence from observational studies.	Can apply to most patients in most circumstances without reservation.
Moderate-quality evidence	RCT with important limitations or strong evidence from observational studies.	Can apply to most patients in most circumstances without reservation.
Low-quality evidence	Observational studies/case studies.	May change when higher-quality evidence becomes available.
Weak recommendation		
High-quality evidence	RCT without important limitations or overwhelming evidence from observational studies.	Best action may differ based on circumstances or patients’ values.
Moderate-quality evidence	RCT with important limitations or strong evidence from observational studies.	Best action may differ based on circumstances or patients’ values.
Low-quality evidence	Observational studies/case studies.	Other alternatives may be equally reasonable.
Insufficient	Evidence is conflicting, of poor quality, or lacking.	Insufficient evidence to recommend for or against.

a This description of supporting evidence refers to therapy, therapeutic strategy, or prevention studies. The description of supporting evidence is different for diagnostic accuracy studies. RCT, Randomized Controlled Trial.

The American Thyroid Association (ATA) created a diagnostic test appraisal system that included consideration of the following methodological elements: consecutive recruitment of patients representative of clinical practice, use of an appropriate reference gold standard, directness of evidence (target population of interest, testing procedures representative of clinical practice, and relevant outcomes), precision of diagnostic accuracy measures (confidence intervals for estimates such as sensitivity and specificity), and consistency of results across studies using the same test that was also used in this guideline^24^ (Table 3, Table 4).

**Table 3:** Interpretation of the American Thyroid Association guideline for diagnostic tests.

Recommendation	Accuracy of diagnostic information versus risks and burden of testing	Implications
Strong recommendation	Knowledge of the diagnostic test result clearly outweighs risks and burden of testing or vice versa.	Patients: in the case of an accurate test for which benefits outweigh risks/burden, most would want the diagnostic test to be offered (with appropriate counseling). A patient should request discussion of the test if it is not offered. In contrast, for a test in which risks/burden outweigh the benefits, most patients should not expect the test to be offered.
		Clinicians: in the case of an accurate test for which benefits outweigh risks/burden, most patients should be offered the diagnostic test (and provided relevant counseling). Counseling about the test should include a discussion of the risks, benefits, and uncertainties related to testing (as applicable), as well as the implications of the test result. In contrast, for a test in which risks/burden outweigh the perceived benefits, most patients should not be offered the test, or if the test is discussed, the rationale against the test should, for the particular clinical situation, be explained.
		Policymakers: in the case of an accurate test for which benefits outweigh risks/burden, availability of the diagnostic test should be adopted in health policy. In contrast, for a test in which risks/burden outweigh the perceived benefits, some restrictions on circumstances for test use may need to be considered.
Weak recommendation	Knowledge of the diagnostic test result is closely balanced with risks and burden of testing.	Patients: most would want to be informed about the diagnostic test, but some would not want to seriously consider undergoing the test; a decision may depend on the individual circumstances (e.g., risk of disease, comorbidities, or other), the practice environment, feasibility of optimal execution of the test, and consideration of other available options.
		Clinicians: different choices will be appropriate for different patients, and counseling about the test (if being considered) should include a discussion of the risks, benefits, and uncertainties related to testing (as applicable), as well as the implications of the test result. The decision to perform the test should include consideration of the patients’ values, preferences, feasibility, and the specific circumstances. Counseling the patient on why the test may be helpful or not, in her/his specific circumstance, may be highly valuable in the decision-making process.
		Policymakers: policymaking decisions on the availability of the test will require discussion and stakeholder involvement.
No recommendation	Balance of knowledge of the diagnostic test result cannot be determined.	Decisions on the use of the test based on evidence from scientific studies cannot be made.

**Table 4:** Recommendations (for Diagnostic Interventions) based on strength of evidence.

Recommendation and evidence of quality	Methodologic quality of supporting evidence	Interpretation
Strong recommendation		
High-quality evidence	Evidence from one or more well-designed nonrandomized diagnostic accuracy studies (i.e., observational—cross-sectional or cohort) or systematic reviews/meta-analyses of such observational studies (with no concern about internal validity or external generalizability of the results).	Implies the test can be offered to most patients in most applicable circumstances.
Moderate-quality evidence	Evidence from nonrandomized diagnostic accuracy studies (cross-sectional or cohort), with one or more possible limitations causing minor concern about internal validity or external generalizability of the results.	Implies the test can be offered to most patients in most applicable circumstances without reservation.
Low-quality evidence	Evidence from nonrandomized diagnostic accuracy studies with one or more important limitations causing serious concern about internal validity or external generalizability of the results.	Implies the test can be offered to most patients in most applicable circumstances, but the utilization of the test may change when higher-quality evidence becomes available.
Weak recommendation		
High-quality evidence	Evidence from one or more well-designed nonrandomized diagnostic accuracy studies (i.e., observational—cross-sectional or cohort) or systematic reviews/meta-analyses of such observational studies (with no concern about internal validity or external generalizability of the results).	The degree to which the diagnostic test is seriously considered may differ depending on circumstances or patients’ or societal values.
Moderate-quality evidence	Evidence from nonrandomized diagnostic accuracy studies (cross-sectional or cohort), with one or more possible limitations causing minor concern about internal validity or external generalizability of the results.	The degree to which the diagnostic test is seriously considered may differ depending on individual patients’/practice circumstances or patients’ or societal values.
Low-quality evidence	Evidence from nonrandomized diagnostic accuracy studies with one or more important limitations causing serious concern about internal validity or external generalizability of the results.	Alternative options may be equally reasonable.
Insufficient	Evidence may be of such poor quality, conflicting, lacking (i.e., studies not done), or not externally generalizable to the target clinical population such that the estimate of the true effect of the test is uncertain and does not permit a reasonable conclusion to be made.	Insufficient evidence exists to recommend for or against routinely offering the diagnostic test.

## Treatment of bilateral sensorineural hearing loss

The use of HAs and CIs early in life has rehabilitated many children, who were able to acquire near-normal language. The effectiveness of these interventions is influenced by factors such as maternal education level, duration of daily device use, and nonverbal intelligence.^9^

Maximizing hearing in each ear optimizes oral language development. Device options for children with bilateral sensory hearing loss include HAs, CIs, and a combination of both (bimodal stimulation).

### Hearing AID

In children with mild-to-moderate bilateral sensorineural hearing loss, the use of HAs should be considered. In the case of severe-to-profound hearing loss, HAs may be insufficient for rehabilitation, and CIs should be considered. Regardless of the degree of hearing loss, HAs should be the first therapy to be considered, even in children with profound hearing loss, before a surgical procedure is indicated. It should be noted that speech assessment and speech therapy are extremely important for children using these devices.

Despite treatment, children with mild-to-severe hearing loss using HAs have poorer receptive and expressive language performance, as well as less vocabulary compared with children with normal hearing.^26^ The worse the hearing deficit, the worse the outcome. Higher maternal education levels and nonverbal intelligence skills, early and prolonged HA use, and greater auditory residual hearing are associated with better linguistic outcomes.

### Cochlear implant

The CI is an implantable prosthesis that provides sound information to patients with severe-to-profound sensorineural hearing loss and no functional gain with HAs. It is the most successful sensory prosthesis in the world.^27^ The CI has an external component (processor) composed of a microphone, speech processor, and stimulator and an internal component composed of a receiver and an electrode array. These components communicate via frequency-modulated waveforms through intact skin.

The sound is captured by the microphone on the external component, selected, and encoded by the processor. The information is then sent to the transmitter, which sends the encoded sounds to the internal component. The electrodes generate electrical impulses that depolarize the auditory nerve fibers.

The cochlear implantation procedure is safe if performed by experienced surgeons. There may be minor (e.g., superficial skin infection, hematoma) or major (e.g., device malfunction requiring revision surgery, facial nerve paralysis, need for device explantation) postoperative complications.^28^ Overall, complication rates reported in the literature range widely from 1% to 5% for major complications and from 4.5% to 15% for minor complications.^29^

Before CIs were made available, only approximately 50% of children with severe-to-profound bilateral hearing loss using HAs were able to acquire spoken language compared with children with normal hearing.^30^ The CI has allowed many children with severe-to-profound bilateral hearing loss to achieve age-appropriate expressive and receptive language skills when they enter elementary school. However, between 30% and 50% of children do not achieve age-appropriate spoken language skills, even in the presence of factors that support successful speech development.^4^, ^31^

Age-appropriate language acquisition in children with CIs has been associated with higher levels of nonverbal intelligence and maternal education, higher levels of residual hearing after surgery, early intervention, a focus on auditory and oral instruction, and the use of novel speech processor technologies.^31^ Although improved school performance and quality of life have been described in children receiving a CI,^32^ CI users have shown long-term educational and occupational levels significantly lower than the population average.^33^

Bilateral implants are the most viable option for verbal language development.^34^ Surgery may be simultaneous or sequential. The interval between the first and second implant procedures must be minimal to allow the development of bilateral skills. Thus, in case of sequential cochlear implantation, the need to quickly implant the second ear (which must occur within 6 months of the first implantation)^35^ should be discussed with the family, and a HA should be used in the nonimplanted ear until surgery.^36^

Several factors influence the frequency with which children with CIs use their device — degree of sensorineural hearing loss, duration of CI experience, age at the time of surgery, and hearing gain with CI.^37^, ^38^ Other important variables include maternal education level, presence or absence of developmental disability, age, and social pressure.^38^

Studies comparing the benefits of bimodal devices and bilateral CIs on oral language skills (receptive or expressive language) suggest that children with profound bilateral hearing loss should undergo bilateral cochlear implantation. Children with thresholds close to 70 dB in one ear and profound hearing loss in the contralateral ear may benefit from bimodal stimulation. However, they should be closely monitored due to the risk of worsening hearing in the ear using the HA.^36^, ^39^

### Cochlear implantation in young children

Early access to hearing is critical for brainstem auditory system development. The lack of auditory stimulation may affect neural development, causing serious long-lasting effects on auditory development, language acquisition, and cognitive skills.^6^, ^40^ Likewise, unilateral auditory stimulation may cause asymmetric development and compromise how the auditory system responds to subsequent CI stimulation in the contralateral ear.^41^ It has been shown that, although early simultaneous bilateral cochlear implantation promotes increased symmetric development of the hearing pathways compared with unilateral or sequential cochlear implantation, cortical processing of sounds still will not be normal.^42^

Better linguistic outcomes may be expected for children who undergo early bilateral implantation.^43^ Therefore, early simultaneous cochlear implantation is likely to be the best intervention to promote oral language development for children with profound hearing loss.^44^ The decision as to whether perform sequential or simultaneous bilateral cochlear implantation is affected by numerous factors and differs according to each health service and country. In Brazil, simultaneous bilateral cochlear implantation has been the standard treatment since 2014 for children with profound deafness.

Additional intraoperative care is required for patients younger than 12 months. Pediatric patients have significantly lower blood volume compared with the adult population. In children younger than 12 months, the total systemic blood volume is approximately 80 mL/kg. Minor blood losses in the pediatric population can have catastrophic effects. Blood losses <10% in this population can lead to hypovolemia.^3^, ^45^ Bleeding from emissary veins and bone marrow oozing from the temporal bone are the main sources of blood loss during pediatric cochlear implantation.^46^ Meticulous hemostasis should be maintained throughout the cochlear implantation procedure in children younger than 12 months to avoid potential complications.

Although the cochlea and facial recess are adult size at birth, changes occur in facial nerve position and mastoid pneumatization during childhood. In children younger than 12 months, there is lateral and superficial displacement of the facial nerve and semicircular canals, as well as underdevelopment of the mastoid tip.^3^, ^47^ To prevent injuries during cochlear implantation in children younger than 12 months, in addition to careful identification of the facial nerve, the incision should not approach the tip of the mastoid.

The skin of children younger than 12 months is significantly thinner than the skin of adults. Musculocutaneous flaps should be manipulated with caution.^4^, ^46^ Skull thickness of younger pediatric patients may be less than a few millimeters. Dura mater exposure during pocket creation in the temporal bone cortex has already been demonstrated to reduce tension on the flap and improve aesthetic outcomes.^48^ However, receptors have become increasingly thinner, rendering this action unnecessary. Given that the skull develops over time and may cause migration of the internal component, the device must be well positioned, and the pocket creation must be well made.^4^, ^46^

#### Recommendations

Regardless of the degree of hearing loss, HAs must be the first therapy to be considered, even in children with profound hearing loss (Strong recommendation — High-quality evidence).

Auditory rehabilitation should be bilateral and conducted as early as possible. Cochlear implantation should preferably be performed before 1 year of age in children with congenital sensorineural hearing loss (Strong recommendation — High-quality evidence).

Children should undergo simultaneous cochlear implantation (Strong recommendation — Moderate-quality evidence).

Cochlear implantation in children younger than 1 year is safe and effective. The rates and nature of major and minor complications are comparable to studies of older children and adults (Strong recommendation — Moderate-quality evidence).

### Unilateral hearing loss

Patients with profound unilateral hearing loss or SSD have sufficient hearing for communication but struggle with sound localization and speech intelligibility in noise. This negatively impacts development and may cause delayed speech acquisition, poor school performance in up to 59% of children, and behavioral changes.^49^

Although the negative implications of SSD are clear, children affected by this condition often have a delay between diagnosis and intervention compared with those with bilateral sensorineural hearing loss.^50^ Late diagnosis may occur due to progressive losses or false negatives in hearing screening. The information conveyed to parents of children with SSD regarding treatment options varies greatly, and physicians often minimize the negative impact of this condition on development, leading to uncertainty in clinical decision-making.^50^ Children with SSD respond to sounds in their better ear and, although language development may be delayed, they often communicate effectively in some situations.^51^

After diagnosis, the school must provide special assistance by placing the child in the front rows, with their better ear facing the teacher. Speech therapy should be conducted if language development is delayed.

In recent years, treatment options for unilateral hearing loss have increased. In addition to HAs for partial losses and Contralateral Routing of Signal (CROS) systems for SSD, other rehabilitation options include Bone-Anchored Hearing Systems (BAHSs) and CIs. The benefit of using a device (implantable or not) should be considered when treating these children.

#### Hearing AIDS and Bone-Anchored Hearing Systems

Importantly, these treatment options do not provide any binaural hearing benefits. This means that speech discrimination in noisy environments and sound localization remain largely impaired.^52^

Traditional options for auditory rehabilitation redirect sound from the affected ear to the normally functioning side. Amplifiers with a CROS system have long been used to redirect sound from the diseased ear to the hearing ear. A microphone is placed on the deaf side to capture sound and transmit it (currently wirelessly) to the receiver on the hearing side. Despite being an option, the use of this device is limited by its high cost. However, using a receiver on the hearing ear may not be an option for some patients, as it disrupts natural hearing acoustics, reducing access to some sounds or modifying monaural level cues on which single-sided listeners become dependent.^53^

BAHSs are an alternative in which a microphone placed on the external processor captures sound and transmits it to the temporal bone and inner ear via bone conduction. This allows the hearing ear to remain open and unoccluded while maintaining monaural spectral cues. In addition to requiring surgery, a limitation of bone conduction stimulation for SSD treatment is the reduced signal level that may occur as it travels through the skull, particularly in high frequencies. BAHSs are more effective than HAs with CROS for sound discrimination in noise and quiet.^54^ Patients may have improved speech discrimination in noise, with results ranging from −3.8 to −4.8 dB signal-to-noise ratio, depending on the location of the sound source.^55^

BAHS users often experience reduced hearing benefit and gain over time. The discontinuation rate of BAHS use over a period of 50 months reached 14%, approximately 3% per year.^56^ The expectations and prejudices of patients with SSD should be established before the BAHS procedure, especially regarding percutaneous systems.

Counseling (habits, work/social environment, expectations) and extensive preoperative testing with a soft band BAHS may help patients understand their expectations. The choice of the most suitable device must be determined by the physician, who will discuss the clinical advantages and disadvantages of the devices with the patient and their family after collecting all qualitative and quantitative data derived from a complete audiologic evaluation.

#### Cochlear implant

The CI is a treatment option for patients with SSD. It restores hearing in the affected year and may provide some hearing benefits that have not been identified with CROS and BAHS use. Patients with SSD using a CI reported improvements in speech perception in the affected ear, hearing in noisy environments, and sound localization compared with those who did not receive any treatment^57^, ^58^ or received other devices.^59^ However, children with congenital SSD may present with anomalies of the eighth cranial nerve, such as hypoplasia or aplasia of the cochlear nerve,^60^ which have been considered contraindications to cochlear implantation.^61^ Thus, MRI should be conducted in the preoperative evaluation of patients with SSD.

Hearing outcomes of CI users with SSD are not the same as those of children with normal hearing. Study outcomes vary greatly, especially due to the duration of auditory deprivation.^12^, ^57^ Speech perception is poorer than normal auditory performance.^62^ Although significantly improved, even in noisy environments,^63^ sound localization is still not completely normal.^64^ Young children typically spend a lot of time in noisy environments, and subtle hearing difficulties may have an extensive negative impact.^65^ In addition, such difficulties may demand a greater listening effort, which, in turn, could lead to higher rates of fatigue and behavioral problems, as seen in some children with SSD.^66^ Likewise, increased listening effort and language problems also occur in children with mild bilateral sensorineural hearing loss.^67^ Improving speech perception in children with SSD by using CIs may reduce listening efforts and assist in language processing. This would explain why children with SSD seem to benefit from early cochlear implantation regarding language development.^68^

A systematic review published in 2020 evaluated 119 children and showed that most of them (79.6%) had improved speech perception in noise after cochlear implantation, which was clinically significant.^11^ However, the same improvement was not observed in all analyzed studies. Cochlear implantation after 4 years of age and high prevalence of hearing loss caused by CMV were associated with negative CI results.

Prolonged auditory deprivation negatively affects CI results. Based on the maturation of the auditory pathway, the estimated sensitive period for treatment is from 3 to 4 years of age.^40^ Early intervention should be conducted during sensitive periods of auditory cortex development.^69^, ^70^ Other hearing solutions have limited utility in pediatric populations and, in most cases, cannot be easily implemented before 5 years of age, when most of the auditory development has already occurred. Unlike redirection (with CROS or BAHS), the CI provides stimulation from both auditory pathways. The CI may serve as an important and beneficial alternative, allowing early intervention and providing additional cues during the critical phase of development, before auditory deprivation causes permanent changes in the CNS.^35^, ^70^

Children with SSD were reported to use their CIs between 6 and 12 h per day, similarly to bilateral CI users.^37^, ^71^ Subjective benefit has been observed between 4-month and 3.5-year follow-ups.^37^ There is evidence that a sensitive period of intervention may play an important role in long-term outcomes and success of cochlear implantation in children with SSD.^70^, ^72^ Animal models point to the negative consequences of untreated asymmetric hearing on auditory development. Electrical stimulation combined with speech therapy may restore the binaural auditory system.^73^

#### Recommendations

Children with asymmetric loss should receive early treatment, given that they develop an aural preference for the hearing ear from an early age, which is difficult to reverse and interrupts their potential to develop binaural hearing (Strong recommendation — Moderate-quality evidence).

Achieving binaural hearing is always the goal, as it is the most physiological path for CNS development (Strong recommendation — Moderate-quality evidence).

BAHSs are more effective than HAs with CROS for sound discrimination in noise and quiet in patients with SSD (Weak recommendation — Low-quality evidence).

Children older than 3-years gain little benefit from cochlear implantation in congenital unilateral hearing loss (Strong recommendation — Low-quality evidence).

The contralateral ear should always be reassessed for the risk of hearing loss (Strong recommendation — Low-quality evidence).

### Auditory neuropathy spectrum disorder

The multidisciplinary team should observe some conditions when treating patients with ANSD. Treatment depends on the severity of the impairment and is defined according to each case. The goal is to restore auditory skills with hearing devices. Among available devices, HAs, frequency modulation or similar systems, and CIs are the most relevant ones. The literature shows an advantage of CIs over HAs.^74^

Some studies have produced different results with cochlear implantation in 25% of cases, which are dependent on patient conditions such as presence of residual hearing, age at the time of cochlear implantation, duration of sound deprivation before cochlear implantation, cognitive aspects, and factors related to socioeconomic status and anatomic morphology, especially of the cochlear and cochlear nerves.^75^, ^76^, ^77^ Patients with ANSD experience significant advantages when using CIs compared with HAs, but they have poorer responses regarding open-set speech perception compared with patients with CIs with a pure tone audiogram of sensorineural hearing loss.^78^

CI users have significantly improved speech perception and auditory performance. Even those with the worst results show a 50% improvement in the recognition of disyllabic words in audiologic tests after cochlear implantation, as well as greater ease of phone use and improved speech understanding in background noise, especially for adults.^79^, ^80^

The CI may provide positive results even when the lesion site seems to involve the auditory nerve. Molecular markers can be of great assistance when making the final treatment decision. The table below lists the genetic markers associated with better or worse CI outcomes in ANSD.

Table 8 shows the genes identified to cause ANSDs and expected CI outcomes (adapted from Shearer et al.)^75^ (see Appendix).

**Fig. 1:**
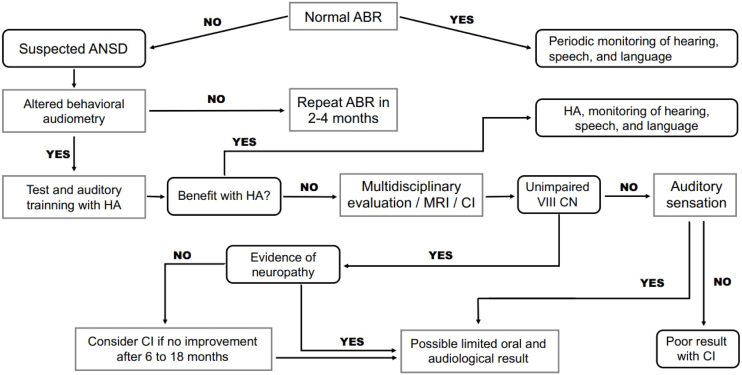
Algorithm for rehabilitation of patients with auditory neuropathy spectrum disorders. ABR, auditory brainstem response; ANSD, auditory neuropathy spectrum disorder; CI, cochlear implant; CN, cranial nerve; HA, Hearing AID.

In addition to the use of hearing devices, auditory training should be conducted and focus on maximizing the signal-to-noise ratio – improving speech perception in noise with or without the use of a frequency modulation or similar system – always providing adequate counseling and defining the limitations that are more closely related to either postsynaptic or CNS lesions. Fig. 1 shows an algorithm for rehabilitation of patients with ANSD.

CI candidates present with variations in auditory nerve function during response to electrical stimulation from the CI. Assessing the integrity of the spiral ganglion and cochlear nerve has become an important focus in predicting CI outcomes. Many studies have attempted to determine whether CI might benefit patients with auditory neuropathy. Auditory neuropathy was initially considered a contraindication to CI. However, after the initial reports of successful cochlear implantation in these patients, larger studies have been conducted and observed that patients with ANSD may benefit from cochlear implantation depending on the lesion site (whether they have neuropathy or synaptopathy).^75^, ^81^

Precisely determining the site of the lesion causing neuropathy, by using molecular diagnosis combined with electrophysiologic data from electrocochleography as well as intraoperative Electrical ABR (EABR), may help predict postoperative CI outcomes and may assist in the final treatment decision.^82^, ^83^ Due to the extreme genetic heterogeneity of deafness, adequate studies require a large number of patients. Thus, any study intended to report results for patients with ANSD and CI must include: (1) a detailed clinical history of the patient, with family history of neuropathy, vision loss, or vestibular disorders. (2) Audiologic data on the degree of hearing loss, age at the time of hearing loss onset, speech recognition tests in quiet and noise. (3) Electrophysiologic data from OAE, ABR, EABR, and electrocochleography (cochlear microphonics, summation potential, auditory nerve neurophonic potential, and adaptation. (4) Implanted CI device and type of stimulation. (5) Post-implantation outcome measures with word recognition scores in quiet and noise. (6) Molecular/genetic data.

Another concerning aspect is that OAE response diminishes over time and eventually disappears in approximately 20%–30% of patients.^84^ Due to the limitations of newborn hearing screening, molecular genetic testing has become an important tool in the screening of newborns, children, and adults who are candidates for cochlear implantation. Several deafness-causing genes have known effects on the mesencephalon and auditory cortex, including DFNB59, CACNA1D, and KCNQ4.^85^

#### Recommendations

Even in patients with mutations affecting the auditory nerve, cochlear implantation may still be recommended due to better hearing outcomes for most patients (Strong recommendation — Moderate-quality evidence).

The lesion site is not the only determinant of CI outcome. In addition to clinical factors such as type and duration of deafness, molecular and physiologic factors that may suggest the status of the cochlea, synapse, spiral ganglion, and auditory nerve should also be considered to provide the best possible outcomes for patients (Strong recommendation — Low-quality evidence).

When conducting the initial evaluation of a patient with suspected auditory neuropathy, the possibility of a molecular diagnosis should be considered in addition to electrophysiologic assessment and therapeutic tests in speech therapy, as well as a multidisciplinary discussion involving speech therapists and geneticists (Strong recommendation — Low-quality evidence).

## Evidence-based treatment of conductive hearing loss

### Children with external/middle ear malformations

Surgical correction of these malformations is one of the most challenging procedures in otolaryngology. The goals of the procedure are to restore hearing and create an external auditory canal that remains patent and infection-free.^86^ However, achieving these goals is often hampered by postoperative complications, such as external auditory canal restenosis (revision surgery may be necessary in 25%–36% of cases). Conductive hearing loss is the most common cause of deafness among these patients, occurring in up to 33% of cases. In addition to these sequelae, peripheral facial paralysis, graft lateralization, and chronic infection may also occur.^87^, ^88^

The Jahrsdoerfer grading system is often used to classify patients before surgery.^89^ In this grading system, patients receive a score of 1–10 according to physical examination and temporal bone CT findings. The preoperative score is correlated with the degree of success that can be expected from the operation. A score of 5 or less disqualifies the patient for surgery, as the risks of surgery outweigh the potential benefits.^90^

The hearing gain varies depending on the severity of the middle ear malformation and the presence of concomitant malformations in the inner ear. Usually, surgical correction is indicated only in patients who have small malformations associated with a favorable anatomy of the temporal bone.^90^ The use of HAs is only possible for patients undergoing surgery who had no external auditory canal stenosis, thus not being a good option. For other patients, there are alternative solutions for hearing rehabilitation, such as implantable systems.^91^

BAHSs represent a revolution in the treatment of patients with middle ear malformations, regardless of the integrity or mobility of the ossicular chain, with a low complication rate. Multiple physiologic mechanisms contribute to hearing via bone conduction. Sound energy is transmitted by skull bone vibrations directly to the cochlea, bypassing the middle ear. Traveling waves propagate along the basilar membrane and stimulate the cochlear nerve. BAHSs can be divided into 4 types:^92^

Nonsurgical — bone vibrators that can be mounted on a behind-the-head band (Baha SoundArc; Cochlear Ltd., Sydney, Australia), elastic band (Softband — Oticon A/S, Smorum, Denmark; Cochlear Ltd., Sydney, Australia), adhesive adapter (ADHEAR; MED-EL, Innsbruck, Austria), teeth (Sonitus SoundBite System), or auditory canal (TransEar).

Percutaneous — Stimulus occurs via a skin-penetrating abutment coupled to a sound processor (Baha Connect — Cochlear BAS, Gothenburg, Sweden; and the Ponto system — Oticon Medical AB, Askim, Sweden).

Transcutaneous — a titanium plate is implanted in the temporal bone and a processor is coupled to a magnet that transmits sound through intact skin (Baha Attract — Cochlear BAS, Gothenburg, Sweden; and Sophono — Medtronic, Jacksonville, FL).

Active transcutaneous — an active implant is placed under the skin and muscles of the temporal bone and communicates with the external sound processor wirelessly via radiofrequency (Bonebridge — MED-EL, Innsbruck, Austria; and Osia2 — Cochlear BAS, Gothenburg, Sweden).

The indication criteria are as follows: (a) unilateral mixed hearing loss: bone conduction threshold — 65 dB (percutaneous), 55 dB (Osia2), and 45 dB (Bonebridge). (b) Speech intelligibility index greater than 60% for open-set recognition of monosyllabic words without HA. (c) Profound unilateral hearing loss: contralateral ear with audiometric thresholds within the normal reference range. (d) Symmetric bilateral conductive or mixed hearing loss: interaural difference between mean bone conduction thresholds at 500, 1000, 2000, and 3000 Hz must not exceed 10 dB and be less than 15 dB at all frequencies. Bilateral implantation is possible. (e) Asymmetric conductive or mixed hearing loss: ideally, BAHS should be placed only unilaterally (on the side with better bone conduction).

Children under 5 years of age or patients with contraindications to surgery should use nonsurgical implant systems, to be decided based on physician and family preferences. From this age onward, BAHSs or active middle ear implants can be used.

The indication of the type of implantable BAHS depends on the experience of the medical team. Advantages of percutaneous systems include rapid surgery (even in malformed skulls), increased bone conduction amplification capacity, few intraoperative complications, and smaller artifacts in MRI which can be performed in up to 3.0 Tesla scanners. However, patients using these systems have aesthetic complaints due to abutment exposure, in addition to the risk of skin complications and osseointegration failure, especially in young children.^92^, ^93^

Active transcutaneous systems have the benefit of keeping the skin intact, being more aesthetically acceptable for patients, and providing good hearing gain, despite being smaller than percutaneous systems. To indicate BAHSs, it is important to note that bone conduction thresholds should be evaluated and that they are normally preserved in patients with microtia. Disadvantages include reduced bone conduction amplification capacity, prolonged operative time, and larger artifacts in MRI which can be performed only in 1.5 Tesla scanners.

Since 2009, the Vibrant Soundbridge® (VSB) hearing device (MED-EL, Innsbruck, Austria) has been used in patients with external or middle ear malformations.^94^ The implanted portion of VSB consists of a receiver coil, a conductive beam, and a Floating Mass Transducer (FMT).^95^ Initially, the FMT was coupled only to the long process of the incus. Currently, new couplers allow the FMT to be placed on the short process of the incus, stapes superstructure, and round window.^96^ VSB model 503 supports up to 1.5 Tesla MRI,^97^ whereas earlier models are incompatible with MRI. Bone conduction thresholds to indicate VSB in conductive hearing loss are 45 dB (500 Hz), 50 dB (1000 Hz), and 65 dB (2000 and 4000 Hz).^96^

Surgery must be performed by experienced otologists and in patients with a favorable anatomy due to the high level of difficulty and risk of serious complications. The usually anomalous split facial nerve configuration makes access difficult (even using intraoperative monitoring), and obliteration of the round window may lead to surgical failure.^98^, ^99^

#### Recommendations

Mastoid CT and MRI are not required early, especially in unilateral malformations (Weak recommendation — Low-quality evidence).

Children under 5-years of age should use nonsurgical implant systems. From this age onward, BAHSs or active middle ear implants can be used (Strong recommendation — Moderate-quality evidence).

The indication of an implantable system should be discussed with the family, which should be informed by the surgeon of the benefits of and contraindications to each system (Weak recommendation — Low-quality evidence).

### Tympanostomy Tube Placement

Tympanostomy Tube Placement (TTP) is one of the most common surgical procedures performed in children. A ventilation tube is placed in the tympanic membrane to ventilate the middle ear. Otitis media is one of the most prevalent conditions in childhood, responsible for a large number of primary care visits.^100^ Over the last 10 years, pneumococcal vaccination has reduced the indications for TTP in children with Acute Otitis Media (AOM).^101^ However, in the United States, in 2014, approximately 9% of children under 17 years of age underwent TTP, in addition to 25%–30% of children with recurrent otitis.^102^

#### Acute otitis media

The mean incidence is 10.8 new episodes per 100 person-years,^18^ but it has declined in high-income countries due to vaccination and adoption of guidelines with strict diagnostic indications.^19^

Treatment includes analgesics, antipyretics, and antibiotics (if necessary). TTP is indicated for management of serious complications, such as mastoiditis, peripheral facial paralysis, meningitis, petrositis, sigmoid sinus thrombosis, and brain abscess.

Recurrent AOM is defined as the occurrence of 3 or more well-documented and distinct episodes of AOM in a 6-month period or 4 or more episodes of AOM in a 12-month period that includes at least 1 episode in the preceding 6-months. TTP should not be performed in children with recurrent AOM without middle ear effusion. However, the American Academy of Otolaryngology-Head and Neck Surgery (AAO-HNS) guidelines include the following exceptions:^103^ (1) children with a history of a severe episode or persistent AOM. (2) Presence of immunosuppression. (3) Presence of AOM-related complications.

TTP may also be an option for children with antibiotic allergy or intolerance.^103^

#### Otitis media with effusion

Otitis Media with Effusion (OME) is defined as the presence of fluid in the middle ear without signs or symptoms of acute infection. Chronic OME occurs when effusion persists for 3-months or longer from the date of onset (if known) or from the date of diagnosis (if onset is unknown).

OME is a common condition, affecting 90% of children before the age of 5-years.^104^ It may occur as a result of Eustachian tube dysfunction, during upper respiratory tract infection, or after the inflammatory process of AOM.^103^ Although OME resolves spontaneously in most cases, up to 40% of patients have repeated episodes, and 10% of episodes last 1-year or longer.^105^ The prevalence of OME is higher in children with Down syndrome or cleft palate.^106^, ^107^

OME persisting for 3-months or longer has been associated with hearing loss, balance disorders, reduced quality of life, and poor school performance.^100^ Given the potential impact of OME on child development, minimizing practice variations in treatment has been a topic of discussion in the latest guidelines. Children younger than 6-months are excluded from almost all guidelines and randomized trials because evidence is extremely limited and their treatment requires individualized decision-making based on specific clinical circumstances.^103^ Children considered at risk for language developmental difficulties are those with a diagnosis such as ASD, blindness or hearing impairment regardless of OME, and syndromic hearing loss, and those with cleft palate or delayed neuropsychomotor development.^103^, ^104^

There is a strong recommendation for the use of pneumatic otoscopy in the diagnosis of OME. In addition, patients with hearing loss, otalgia, or both should be assessed with pneumatic otoscopy to rule out OME.^100^ However, equipment is not readily available in the offices. Diagnosing the presence of chronic OME is imperative due to its negative effect on auditory and speech development and on the tympanic membrane, potentially leading to the formation of tympanic retraction pockets and cholesteatoma.^108^

If the diagnosis remains uncertain after pneumatic otoscopy, tympanometry should be performed (strong recommendation).^103^, ^108^ At-risk children should be identified and evaluated for the presence of OME. This evaluation should be repeated at 12 and 18 months of age. However, if healthy children present with OME, it is strongly recommended that they be reevaluated after 3-months from the diagnosis of OME given the natural history of OME spontaneous resolution. Up to 90% of OME cases resolve spontaneously within 3-months.^103^

Current guidelines do not recommend the use of systemic or topical intranasal steroids, antibiotics, antihistamines, or decongestants in the treatment of OME due to a lack of evidence showing their effectiveness compared with placebo.^103^, ^109^, ^110^ However, patients with concomitant allergic rhinitis may benefit from intranasal steroids, which may decrease the inflammatory component in the airway that contributes to the presence of OME.^111^ Although an association between allergy and OME has been suggested, evidence of a direct causal relationship is lacking.^19^ Maneuvers such as Valsalva can help unclog the Eustachian tube, but evidence is still lacking.^108^

The latest AAO-HNS recommendations for TTP in children aged 6-months to 12-years were published in 2022.^103^ Middle ear effusion is common in children after upper respiratory tract infections. The increased risk of infections in young children is partly explained by their immature immune system and Eustachian tube ventilation dysfunction caused, in part, by its more horizontal position.^112^ The AAO-HNS guidelines state that OME of less than 3-months’ duration does not qualify as an indication for TTP.^103^ Exception includes previously defined at-risk children.

An observational surveillance interval of 3–6-months should be established in children with OME in order to identify one of the following disease courses: (a) OME resolves spontaneously. (b) Hearing loss is detected. (c) Abnormalities of the tympanic membrane or middle ear are suspected or observed.

This surveillance interval allows clinicians to reevaluate children for surgical intervention, avoids complications, and allows for ongoing family counseling. Pediatricians should be aware of these recommendations to ensure adequate treatment for children.

Age-appropriate hearing evaluation should be performed when a child becomes a candidate for TTP. Establishing the auditory threshold and identifying the presence of a concomitant sensorineural component helps clinical decision-making about the need for surgery. Children with ventilation tubes should be reevaluated every 3-months.

According to the AAO-HNS guidelines, indications for TTP include^103^: (a) bilateral OME for 3-months or longer with documented hearing loss. (b) Unilateral or bilateral OME for 3-months or longer with symptoms that include ear discomfort, vestibular problems, behavioral issues (including poor school performance), and reduced quality of life. (c) Recurrent AOM and unilateral or bilateral OME at the time of assessment. (d) At-risk children with unilateral or bilateral OME who are believed to have a low probability of spontaneous resolution, or the presence of OME for 3-months or longer.

When TTP is recommended for children under 4-years of age, adenoidectomy should not be performed simultaneously unless there is a clear indication for this surgical procedure, such as chronic adenoiditis or nasal obstruction caused by adenoid hypertrophy. However, in children aged 4-years or older, adenoidectomy with TTP is recommended for the treatment of OME.^103^, ^108^ Adenoidectomy has shown a protective effect only in children older than 4-years.^113^

TTP has been shown to be effective at reducing the prevalence of chronic OME, improving hearing and positively affecting child quality of life.^103^

Several ventilation tubes are available on the market, and the choice of tube type should be made in the clinical context of the treatment strategy for the individual child. Ventilation tubes such as Paparella can extrude within 6–12 months, compared with Armstrong tubes which generally extrude within 9–14 months. Long-term tubes are those lasting up to 2 years.^108^ The clinician should not place long-term tubes as initial surgery for children who meet criteria for TTP unless there is a specific reason based on a need for prolonged middle ear ventilation.

Perioperative education and family counseling are essential in the care of these patients and allow caregivers to assess the expected duration of tube function, recommended follow-up schedule, and detection of complications.^103^ One of the most common complications is otorrhea (generally transient), occurring in up to 26% of children with a ventilation tube.^114^ In uncomplicated cases, the use of ear drops alone is recommended. Local drug concentration levels in ear drops are 1000 times higher than those of the same oral antibiotic.^108^ These medications are effective against *Pseudomonas aeruginosa* and penicillin-resistant *Staphylococcus aureus*, with systemic antibiotics being limited to immunocompromised children or those with acute complications, such as cellulitis of adjacent skin and concurrent bacterial infection of the paranasal sinuses or pharynx. However, ear drops should not be used indiscriminately.

Other reported complications of TTP are obstruction (7%), premature extrusion (4%), granulation tissue (4%), and displacement to the middle ear (0.5%). Myringosclerosis and the associated minimal effects on hearing may account for the long-term sequelae of TTP. Tympanic membrane perforations, which may require repair, are observed in a mean of3% of children after TTP.^115^

Water precautions in children with ventilation tubes should not be routinely encouraged unless there is otalgia from water exposure (river, pond, or swimming pool), deep sea diving, or presence of otorrhea.^116^

#### Recommendations

Children with OME should be evaluated periodically. TTP is not indicated for all cases with a less than 3-month diagnosis (Strong recommendation — Low-quality evidence).

Adenoidectomy is not indicated for children under 4-years of age unless there are respiratory symptoms (Strong recommendation — Low-quality evidence).

Patients with recurrent AOM have no indication for routine TTP (Strong recommendation — Moderate-quality evidence), with exceptions.

There are no swimming restrictions for children with ventilation tubes (Strong recommendation — Low-quality evidence).

The use of ear drops in children with ventilation tubes should include careful consideration, and they cannot be routinely used (Strong recommendation — Low-quality evidence).

### Tympanoplasty in children with simple chronic otitis media

Approximately 98.7 million people have otitis media-related hearing loss, according to the World Report on Hearing,^20^ which shows an incidence of chronic otitis media of 4.76%, approximately 30 million cases/year, and a prevalence of approximately 200 million cases globally. Of these, 22.6% occur in children under 5-years of age. Otitis media is one of the main causes of hearing loss in childhood and, although the incidence of infections decrease with age, hearing loss can persist.

Chronic suppurative otitis media leads to some degree of hearing loss in 50% of cases due to tympanic membrane perforation and changes in the ossicular chain (conductive hearing loss), hair cell damage caused by recurrent infections (sensorineural hearing loss), or mixed.^21^

In cases of chronic otitis media with tympanic membrane perforation, tympanoplasty with or without ossicular reconstruction is the treatment of choice and aims to eradicate the infection, close the tympanic perforation, and restore hearing if possible. A systematic review showed that there is improvement in hearing acuity for 7 in every 10 patients undergoing tympanoplasty.^117^ Surgical success rates in adults range from 60% to 99%, whereas in children these rates range from 35% to 94%,^21^ opening up a discussion on which factors would be related to the surgical prognosis: Age; Site of perforation; Contralateral ear; Otorrhea; Graft type; Surgical technique.

#### Age

Immune system immaturity, Eustachian tube angle and length, recurrent upper respiratory tract infections, and postoperative care are factors suggesting that age may be associated with the surgical success of tympanoplasty, but no statistically significant difference has been observed when comparing ages 4–16 years.^21^, ^118^, ^119^, ^120^

#### Site of perforation

Although anterior perforations have been considered a poor prognostic factor, due to low vascularization and more difficult access, recent studies have produced different results and no longer report the site of perforation as a determinant of poorer outcomes.^21^, ^118^

#### Contralateral ear

Because Eustachian tube function is difficult to measure, assessment of the contralateral ear has been suggested for its evaluation. A normal contralateral ear is a good prognostic factor for the surgical outcome of tympanoplasty, but when altered with tympanic membrane perforation or retraction, it is not necessarily indicative of poor prognosis.^21^, ^119^ In these cases, tympanoplasty should be deferred until the age of 7-years, when the anatomy of the Eustachian tube becomes more satisfactory.^121^

#### Otorrhea

Preoperative dry ear time has long been debated as a prognostic factor in tympanoplasty. Some authors have reported better results with dry ears within at least 3-months of surgery, whereas others have observed no difference with wet ears for a period closer to the procedure date.^21^, ^118^, ^121^

#### Graft type

Because cartilage grafts are often thick, their use may be disregarded due to the possibility of not improving or worsening hearing acuity, although this fact has not been observed. The use of temporalis fascia, perichondrium or cartilage grafts has produced no significant difference in the results of perforation closure or audiometry.^21^, ^119^, ^121^ Because cartilage is thicker and tends to resist resorption, it should be the preferred graft material in cases where there are signs of an acute inflammatory process intraoperatively.^121^

### Surgical technique

#### Underlay, overlay, or inlay

When comparing the surgical results for closure of a tympanic membrane perforation and the air-bone gap, there is no significant difference in the choice of lateral (overlay), medial (underlay), or perforation (inlay) graft placement.^21^, ^119^ However, in cases where only myringotomy is indicated, with no need for a tympanomeatal flap, the use of a butterfly cartilage graft in the inlay technique has shortened operative time and reduced procedure-related costs by 65%.^122^

#### Endoscopic vs microscopic

The use of endoscopic approaches in ear surgery has become increasingly common, leading to less invasive procedures with transcanal access and reducing recovery time and, sometimes, procedure-related costs. Another benefit is superior visualization of the tympanic cavity, enabled by the use of wide-angle lenses. However, the unique anatomic features of the pediatric ear should be considered. Although the tympanic membrane in children is adult size at birth, its angle and the length and size of the internal acoustic meatus differ significantly.^123^ Regarding the surgical results for both perforation closure and audiometry, no differences have been observed between the use of microscopic and endoscopic tympanoplasty in children.^123^, ^124^

#### Hearing threshold

The impact of hearing loss and the potential complications resulting from recurrent middle ear infections are well known, especially in childhood. For these reasons and in view of the available literature, it can be concluded that the treatment of chronic otitis media with tympanic membrane perforation (tympanoplasty with or without ossicular reconstruction) should be started as soon as possible, taking into account the age of the child, status of the ear and Eustachian tube, and the technique with which the surgeon feels most comfortable.

#### Recommendations

There is no level of evidence to contraindicate tympanoplasty considering only the age of the child (Insufficient).

Tympanoplasty performed in subtotal perforations or in children with an altered contralateral ear are more likely to fail (Weak recommendation — High-quality evidence).

There is no consensus on the time required for dry ear before indicating surgery (Insufficient).

The type of graft used (inlay cartilage vs underlay temporalis fascia) does not change the outcome of tympanoplasty (Strong recommendation — High-quality evidence).

## Conclusion

Treatment may include only speech therapy in children with a normal audiogram who present only with a delay in speech development. In children with hearing loss, in addition to speech therapy, HAs or implantable systems may be indicated. Even in children with profound hearing loss, both the use of HAs and behavioral assessments while using the device are important.

Children with unilateral hearing loss deserve special attention and should be treated accordingly. This document has provided an overview of the problems that unilateral hearing loss can cause and the importance of binaural hearing. It is up to the medical team to decide whether to refer a patient with profound congenital deafness for cochlear implantation, BAHS, or HA with CROS, but it is important to emphasize that cochlear implantation has better outcomes if performed early. Therefore, even in children with mild-to-moderate unilateral sensorineural hearing loss, HAs should be offered through the Brazilian public health system to prevent CNS alterations that might be permanent.

Considering that children with hearing loss, regardless of severity, may also have visual impairment, early ophthalmologic evaluation is imperative.

Table 5 shows treatment recommendations for children according to the type and severity of hearing loss.

**Table 5:** Treatment recommendations for children with hearing loss.

Hearing loss	Severity	Treatment	Notes
Bilateral sensorineural	Mild	HA	
	Moderate	HA	
	Severe	HA or CI	
	Profound	HA or CI	
Unilateral sensorineural	Mild	HA	
	Moderate	HA	
	Severe	HA/CROS system/BAHS/CI	
	Profound	CROS system/BAHS/CI	If indicated, CI surgery should be performed early in congenital loss.
Conductive/mixed	Microtia/EAC stenosis	Age <5 years — non-implantable BAHS	
		Age ≥5 years — implantable BAHS	

HA, Hearing AID; CI, cochlear implant; CROS, Contralateral Routing of Signal; EAC, external auditory canal; BAHS, Bone-Anchored Hearing System.

## Funding

The authors have no financial relationships relevant to this article to disclose.

## Conflicts of interest

The authors declare no conflicts of interest.
